# Pro-inflammatory effect of obesity on rats with burn wounds

**DOI:** 10.7717/peerj.10499

**Published:** 2020-12-08

**Authors:** Chan Nie, Huiting Yu, Xue Wang, Xiahong Li, Zairong Wei, Xiuquan Shi

**Affiliations:** 1 Department of Epidemiology and Health Statistics, School of Public Health, Zunyi Medical University, Zunyi, Guizhou, China; 2 Department of Epidemiology and Health Statistics, School of Public Health, Guizhou Medical University, Guiyang, Guizhou, China; 3 Burns & Plastic Surgery, Affiliated Hospital of Zunyi Medical University, Zunyi, Guizhou, China

**Keywords:** Obesity, Burn, Chemokine, Cytokine, Growth factor

## Abstract

**Objective:**

A burn is an inflammatory injury to the skin or other tissue due to contact with thermal, radioactive, electric, or chemical agents. Burn injury is an important cause of disability and death worldwide. Obesity is a significant public health problem, often causing underlying systemic inflammation. Studying the combined impact of burn injuries on obese patients has become critical to the successful treatment of these patients. The aim of this paper is to highlight the effect of inflammation associated with burn injuries on several body weight group in a rat study.

**Materials and methods:**

Different degrees of obesity and burns were established in rats and divided into a normal weight group, overweight group, obese group, second-degree burn group, third-degree burn group, over-weight second-degree burn group, over-weight third-degree burn group, obese second-degree burn group, and obese third-degree burn group (20 rats per group). Changes in inflammatory factors and growth factor were measured on the 1st, 3rd, 7th and 14th days after burns were inflicted.

**Results:**

The ELISA test showed that in the unburned control group, MCP-1, IL-1β and TNF-α protein expressions in the obese and over-weight groups were higher than the normal-weight group (*P* < 0.05). RT-PCR test showed that the expressions of MCP-1, IL-1β and TNF-α genes in the obese group were higher compared to the overweight and normal weight groups (*P* < 0.05). Three and 7 days after burns were inflicted, the level of VEGF in the normal weight group was higher than the obese group (*P* < 0.05), however increased VEGF was not observed on days 1 and 14.

**Conclusion:**

Burn injury and obesity have a mutually synergistic effect on the body’s inflammatory response.

## Introduction

Burns are one of the most devastating types of injury. Burns can cause abnormal metabolism and various inflammatory reactions in the body, which lead to skin or other organ damage, disability, and death (Nie et al., 2019; Stone et al., 2018; Tian et al., 2018). According to the World Health Organization (WHO), there are nearly 180,000 people dying from burns each year, especially in low- and middle-income countries (Smolle et al., 2017). In Bangladesh, Colombia, Egypt and Pakistan, approximately 18% of burned children were left permanently disabled.

Obesity is a chronic metabolic disease characterized by excessive accumulation of body fat. In the past few decades, the concept of obesity as simply depositing lipids has been replaced by a new hypothesis that it is a kind of chronic systemic inflammatory disease (Trayhurn & Wood, 2004; Wellen & Hotamisligil, 2003). Savetsky et al. (2014) found that obese patients have a higher risk of lymphedema due to an impaired baseline lymphatic clearance and an increased tendency toward inflammatory response syndrome when injured.

Obesity has become a critical factor in the medical treatment of burns, as many patients also have diseases such as diabetes. Studies show that obesity is a risk factor that increases hospitalization time, infection rate, and mortality in burned children (Ghanem et al., 2011; Patel et al., 2010; Ross, Burris & Murphy, 2014). The number of obese burn patients who were hospitalized for more than 7 days was reported to be 4.1 times greater than non-obese burn patients and obese burn patient mortality was 2.6 times greater than non-obese burn patients (Carpenter et al., 2008). Although Kraft et al. (2012) stated that obesity affects patients’ metabolic function without increasing the infection rate, organ failure rate and mortality of burnt patients; Ray et al. (2015) concluded that obesity increased the wound infection rate and length of hospital stay, but the mortality rate was reduced. However, the manner in which the etiology of obesity affects the prognosis of burns is still unclear.

Adipose tissue is mainly constituted by adipose cells, that not only store energy, but also have an active endocrine role in the secretion of biomolecules known as adipokines, including monocyte chemoattractant protein 1 (MCP-1), interleukin-6 (IL-6), leptin, and cytoinflammatory factors such as tumor necrosis factor (TNF- *α*) (De Lorenzo et al., 2016; Manna & Jain, 2015). MCP-1 is mainly secreted by monocytes, macrophages, fibroblasts, or vascular endothelial cells, and can specifically activate macrophages to promote inflammation in the body (Cranford et al., 2016; Panee, 2012). Studies have shown that MCP-1 is significantly increased in the adipose tissue of obese patients, and that over-expressed MCP-1 causes circulating monocytes to aggregate into adipose tissue (Boutens & Stienstra, 2016; Remmerie, Martens & Scott, 2020). Monocytes differentiate into macrophages and produce additional inflammatory cytokines, causing further inflammation (Boutens & Stienstra, 2016; Remmerie, Martens & Scott, 2020). Cheng (2016) found that the expression of serum MCP-1 in obese patients was higher than that in healthy people, and the level of MCP-1 decreased after controlling diet to decrease body weight. Interleukin-1 (IL-1) and TNF-α are mainly produced by mononuclear macrophages stimulated by lipopolysaccharide (LPS), which are involved in the regulation of immune and inflammatory systems, and are important inflammatory cytokines produced after burns. IL-1 and TNF-α can stimulate MCP-1 secretion in various types of cells such as vascular smooth muscle cells, endothelial cells and adipocytes, while MCP-1 itself can induce mononuclear macrophages to express the above inflammatory factors in a complementary relationship (Cranford et al., 2016; Panee, 2012; Arcidiacono et al., 2020). Vascular endothelial growth factor (VEGF) is an important member of the platelet-derived growth factor family and has the function of promoting endothelial cell proliferation. It is released and activated following trauma or other tissue damage, playing an important role in promoting tissue repair (Behm et al., 2012). Therefore, we hypothesized that obesity might have a pro-inflammatory effect in a study of rats with burn wounds.

To test this hypothesis, this study intends to establish a relationship between obesity and burn wounds through animal experiments. The study also aims to explore inflammatory factors like MCP-1, IL-1, TNF-α and growth factor VEGF in rats with different burn severities at different levels of obesity. Finally, the risk of obesity on the prognosis of burns will also be evaluated.

## Materials and methods

### The obesity model

Three-week-old male Sprague Dawley (SD) rats, weighing 70-100g, were purchased from the Animal Experimental Center (production license number: SCXK 2012-0005). All animals were housed at a standard temperature (22 ± 1 °C), humidity (65%–70%) and in a 12 h light-dark cycle with food and water provided ad libitum. Following overnight fasting, rats were sacrificed under anesthesia with 1% sodium pentobarbital (40 mg/kg). Institutional animal care and use committee of Zunyi Medical University provided full approval for this research (No. [2015]2-003).

Sixty rats were fed a regular diet and another rats group was fed a high-fat diet (HFD). The HFD feed was processed by the Animal Experimental Center of the Army Military Medical University. The HFD feed formula was: 60% of basic feed, 10% of lard (cooked), 5% of sucrose, 5% of whole milk powder, 8% of peanut, 10% of egg, and 2% of salt (Chandler et al., 2005; Wang et al., 2019). The weight of the rats was monitored and recorded weekly. The criteria for selecting overweight and obese rats was to select HFD group rats who increased their body weight by 10% or 20%, respectively, compared to the average weight of the regular diet group (Chandler et al., 2005; Wang et al., 2019). In our study, we selected 60 obese rats and 60 overweight rats.

After eight weeks, as the rat body weights reached the maturity, the body length (distance from the tip of the nose to the anus) was recorded and Lee’s index (weight (g)^1∕3^ ×10^3^/body length (cm)) was calculated (Bernardis & Patterson, 1968; Miao et al., 2019).

### The burn model

The SD rats were anesthetized by intraperitoneal injection of 1% sodium pentobarbital (40 mg/kg), and the dorsum was shaved and then depilated with sodium sulfide (8%), confirming that the skin after hair removal was not damaged. A burn device was prepared from a tabletop constant temperature burner (YLS-5Q, ZS Dichuang Crop., Beijing, China). Two pairs of symmetrical 2.5 cm^2^ burn wounds were made on both sides of the spine at 500g pressure (Venter, Monte-Alto-Costa & Marques, 2015). Rats were anesthetized with ether to alleviate their pain. Hematoxylin and eosin (HE) staining indicated that a burn of 80 °C for 8 s and a burn of 100 °C for 10 s can cause second and third-degree burns respectively.

### Experiment design

**Figure 1:**
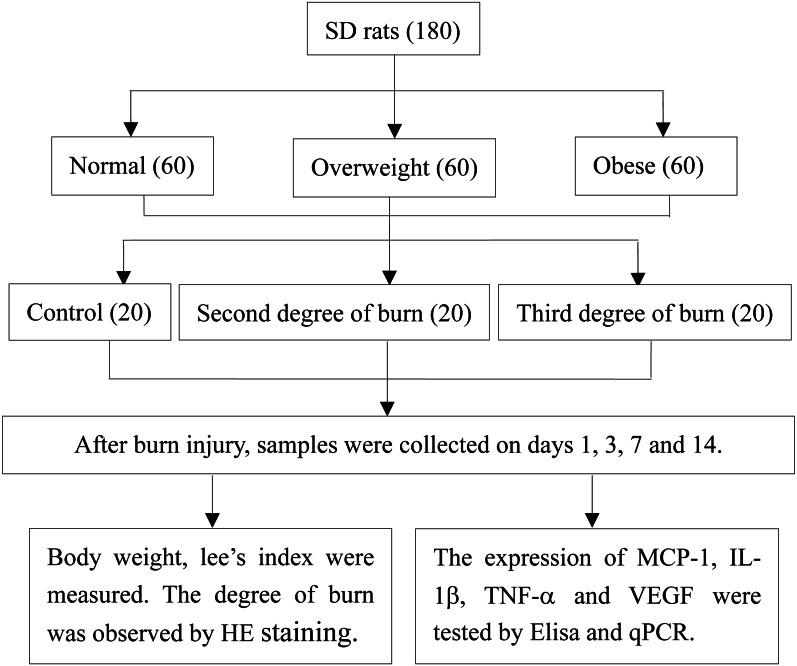
Study design.

After successfully inflicting a standard burn on the different diet groups, the rats were divided according to a 3*3 factorial design. Each of the weight groups (obese, overweight and normal weight) were randomly divided into the following: the normal weight group, overweight group, obese group, second-degree burn group, third-degree burn group, overweight second-degree burn group, obese second-degree burn group, overweight third-degree burn group, and obese third-degree burn group. Each group had 20 rats. The high fat or normal feeds were continued and no dressing was used following the burn injuries. Body weight was observed and recorded on the 1st, 3rd, 7th and 14th day after burns were inflicted. A control group with no burn injury was depilated following the same method as the above diet groups (Fig. 1).

### Sample collection and testing

According to the prognosis of burns, tissue and blood samples were collected on days 1, 3, 7, and 14 after burns were inflicted (Hongjie et al., 2009; Valvis et al., 2015). The blood was collected for serum extraction, and the wound tissue was collected for RNA analysis and fixed in 10% formalin for 24 h for HE staining. The enzyme-linked immunosorbent assay (ELISA) was performed to test the protein expression of MCP-1, IL-1 *β* and TNF-α in the serum. The ELISA kit was from Shanghai Jianglai Biological Technology Co., Ltd., China. The experimental procedure was conducted according to the manufacturer’s protocol. Real-time PCR was performed to test the mRNA expression of MCP-1, IL-1β, TNF-α and VEGF genes in burned tissue. The PCR reagents used were Prime Script RT Master Mix (Perfect Real Time) (RR036A, TAKARA) and TB Green Premix Ex Tap II (Tli RnaseH Plus) (RR820A, Takara, Japan). Total RNA extraction and the Real-Time PCR Detection System (CFX96, BIO-RAD, USA) was performed according to the manufacturer’s protocol. The mRNA expression of each gene in burned tissue was normalized using the average expression of *β*-actin protein genes and comparing that with the data obtained from the control group using the 2^−(ΔΔ*CT*)^ method (Livak & Schmittgen, 2001).

### Statistical analysis

SPSS18.0 software was used for data analysis. Difference comparisons were analyzed by two-way ANOVA, and the least significant difference (LSD) method was used for multiple comparison between groups. The interaction effect was analyzed by factorial analysis. *P*-values <0.05 were considered statistically significant.

## Results

### Body weight and Lee’s index in different weight groups

The body weight and Lee’s index in the overweight and obese groups were significantly higher than the normal weight group (*P* < 0.05). Body weight and Lee’s index in the obese group were statistically higher than the overweight group (*P* < 0.05) (Table 1).

**Table 1:** Comparison of body weight and Lee’s index between different weight groups (mean ± SD).

Group	N	Weight (g)	Lee’s index
normal weight	60	386.43 ± 30.98	304.55 ± 11.87
overweight	60	451.75 ± 21.59^a^^b^	308.30 ± 8.76^a^^b^
obese	60	497.43 ± 34.91^a^	313.67 ± 10.02^a^

**Notes.**

a Compared with the normal group, *P* < 0.05.

b Compared with obese group, *P* < 0.05.

### The HE staining images displayed different burn severity in wounds

**Figure 2:**
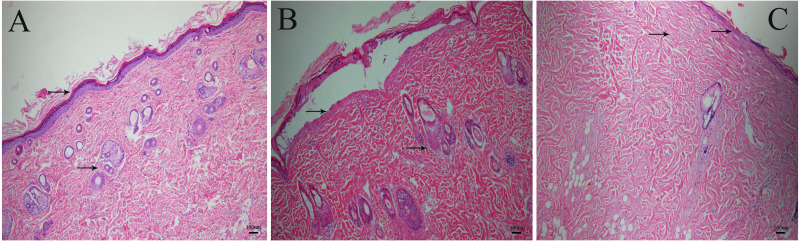
The HE staining images of burned rat tissue. (A) Normal skin tissue. (B) Wound burned at 80 °C for 8 s, with a damaged epidermis, but not involving the dermis. (C) Wound burned at 100 °C for 10 s, with fused collagen bundles, and destroyed hair follicle and sebaceous glands.

As is shown in the Fig. 2, inflicting burns at 80 °C for 8 s resulted in second-degree burns. There was complete necrosis in the epidermis, blister formation, but no damage to the dermis. The texture of the collagen bundle was clear, and the skin attachments such as the hair follicle and sebaceous glands were still alive. Inflicting burns at 100 °C for 10 s resulted in third-degree burns. There was complete necrosis in the epidermis, collagen bundles were fused, the texture was not clear, burns involved the dermis and the attachments of the hair follicle and sebaceous glands were destroyed (Fig. 2).

### Changes in weight after burn injuries

In the unburned group control group, the body weight in obese and over-weight groups increased on each subsequent sampling day, and was greater compared to the normal weight group (*F* = 6.302, *P* = 0.013; *F* = 42.534, *P* < 0.001; *F* = 19.48, *P* < 0.001; *F* = 16.791, *P* < 0.001). The severity of a burn had no statistically significant effect on the weight of rats.

### The effects of obesity and burn on the protein expression of MCP-1, IL-1β and TNF-α in blood serum

Factorial analysis showed that obesity and burn severity had no interactive effect on MCP-1expression. According to the main effect analysis, on day 14 the level of MCP-1 was greatest in the control group, and the second-degree burn group had greater expression than the third-degree burn group (*F* = 9.406, *P* < 0.05). On days 1 and 3, the levels of MCP-1 in the over-weight and obese groups were higher than that in normal weight group (*F* = 10.482, *P* < 0.05; *F* = 7.079, *P* < 0.05) (Table 2).

**Table 2:** The effect of burns and obesity on the expression of MCP-1, TNF-α and IL-1β protein.

	Factor	Test days	df	*F*	*P*
MCP-1	Burn severity	1	2	0.812	0.451
		3	2	0.835	0.441
		7	2	0.505	0.608
		14	2	9.406	0.000
	Degree of obesity	1	2	10.482	0.000
		3	2	7.079	0.002
		7	2	0.249	0.781
		14	2	2.983	0.062
TNF-α	Burn severity	1	2	2.687	0.080
		3	2	1.464	0.243
		7	2	1.034	0.365
		14	2	0.283	0.755
	Degree of obesity	1	2	2.549	0.091
		3	2	1.910	0.161
		7	2	8.065	0.001
		14	2	2.222	0.122
IL-1β	Burn severity	1	2	0.087	0.917
		3	2	1.645	0.206
		7	2	2.260	0.118
		14	2	0.249	0.781
	Degree of obesity	1	2	0.101	0.904
		3	2	0.636	0.535
		7	2	0.718	0.494
		14	2	0.046	0.956

**Notes.**

Factorial analysis showed that obesity and burn severity had no interactive effect on MCP-1, TNF-α and IL-1β expression.

The interaction between Burn severity * Degree of obesity was therefore not included in the analysis. The main effects were from factors of Burn severity and Degree of obesity separately.

Factorial analysis showed that obesity and burn severity had no interactive effect on IL-1β expression. The two-factor main effect analysis also showed no statistically difference. The effect of the weight factor alone showed that the expression of IL-1β was significantly higher in the obese group compared to the normal weight group (*F* = 3.464, *P* = 0.038) (Table 2).

Factorial analysis showed that obesity and burn severity had no interactive effect on TNF-α expression. According to the main effect analysis, the expression of TNF-α was not statistically different across groups of varying burn severity. On day 7, the expression of TNF-α protein was highest in the obese weight group, and the overweight group had higher expression than the normal weight group (*F* = 8.065, *P* < 0.05) (Table 2).

### Gene expression in wounds

#### The effect of obesity and burns on the gene expression of MCP-1

The detection of MCP-1, IL-1β, TNF-α and VEGF genes in skin wounds were determined by RT-PCR relative quantification. The unburned control group was included in this research as a baseline reference, so the control groups are not included in the analysis.

**Figure 3:**
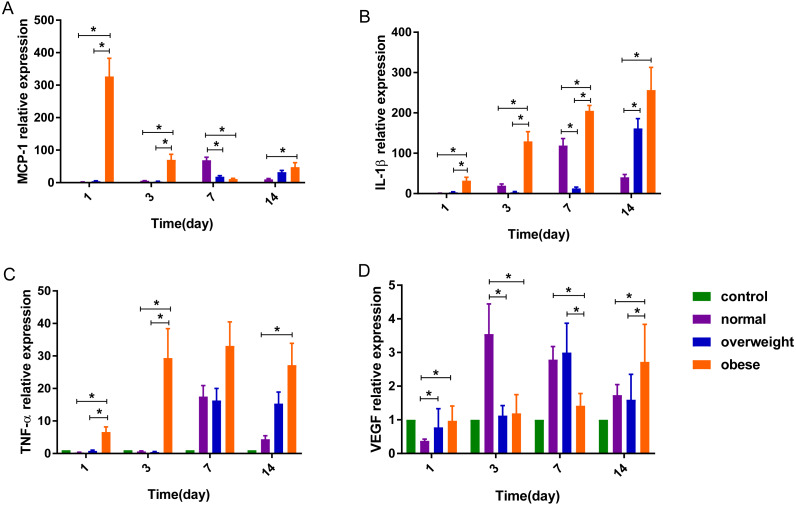
The expression of (A) MCP-1, (B) IL-1β, (C) TNF-α and (D) VEGF genes in rats with different degrees of obesity after burns were inflicted (^∗^*P* < 0.05).

**Figure 4:**
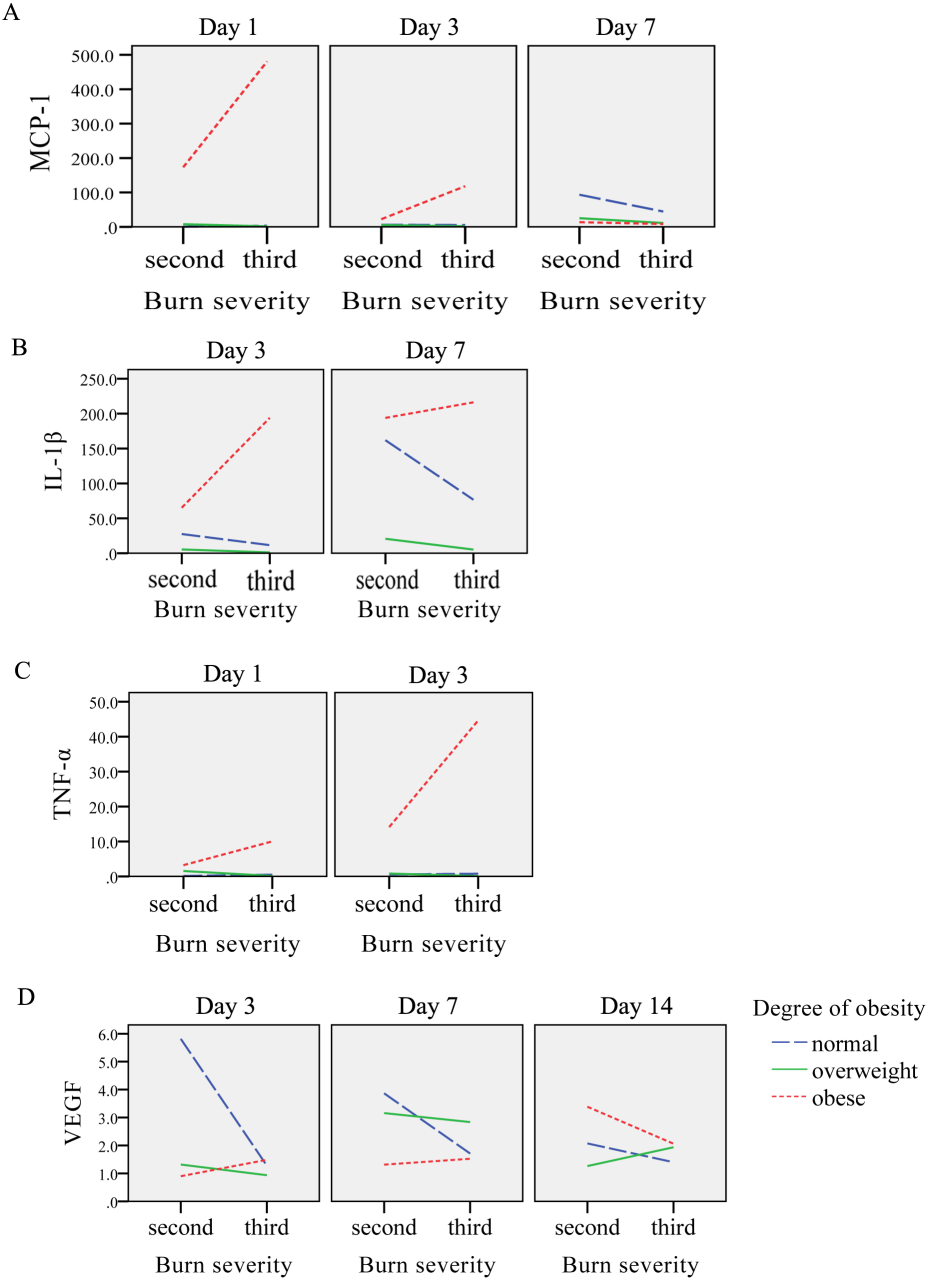
The interaction diagrams between burn severity and obesity on (A) MCP-1, (B) IL-1β, (C) TNF-α and (D) VEGF.

On the 1st day after burns were inflicted in the obese group, the relative expression of MCP-1 gene was at its highest, gradually decreasing after day 1. In the overweight and normal weight groups, the level of MCP-1 increased gradually over days 1, 3 and 7, with the highest expression on day 7 and 14, respectively. Factorial analysis showed that burns and obesity factors had an interactive effect on the expression of MCP-1 gene on days 1, 3 and 7. On the 1st, 3rd and 14th day after burns were inflicted, the level of MCP-1 in the obese group was higher than the normal and over-weight groups (*P* < 0.05), and severe burns had a tendency to increase the expression of MCP-1 in the obese group. On day 7, the level of MCP-1 in the obese group was slightly lower than that in over-weight group, and lower than that in the normal weight group. The mean level of MCP-1 was highest in the obese group, with the normal weight group having higher expression than the overweight group (*P* < 0.05) (Table 3, Figs. 3A, 4A).

**Table 3:** The effect of burns and obesity on the relative expression of MCP-1, TNF-α and IL-1 *β* protein.

Factor	Test days	df	MCP-1		IL-1β	
			*F*	*P*	*F*	*P*
Burn severity	1	1	44.596	0.000	0.388	0.539
	3	1	55.761	0.000	20.777	0.000
	7	1	35.586	0.000	5.191	0.032
	14	1	2.565	0.122	2.026	0.168
Degree of obesity	1	2	202.592	0.000	12.081	0.000
	3	2	115.579	0.000	100.153	0.000
	7	2	91.835	0.000	93.771	0.000
	14	2	4.948	0.016	9.092	0.001
Burn severity^*^ Degree of obesity	1	2	46.152	0.000	1.119	0.343
	3	2	64.370	0.000	34.227	0.000
	7	2	12.120	0.000	7.535	0.003
	14	2	0.357	0.704	0.206	0.815

**Notes.**

Factorial analysis showed that obesity and burn severity had no interactive effect on MCP-1, TNF-α and IL-1β expression. The interaction between Burn severity * Degree of obesity was therefore not included in the analysis. The main effects were from factors of Burn severity and Degree of obesity separately.

#### The effect of obesity and burn on the gene expression of IL-1β

After burns were inflicted, the relative expression of IL-1β gene in overweight and obese groups increased gradually, and peaked on day 14. The normal weight group had highest expression on day 7. Factorial analysis showed that burn and obesity factors had an interactive effect on the expression of IL-1β gene on days 3 and 7, when the level of IL-1β gene in the obese group was higher than that in the overweight and normal weight groups (*P* < 0.05). Severe burns had a tendency to promote the expression of IL-1β in the obese group, and a trend of decreased expression was observed in normal and overweight groups. The mean level of IL-1β was highest in the obese weight group, with the overweight group expressing more than the normal weight (*P* < 0.05) (Table 3, Figs. 3B, 4B).

#### The effect of obesity and burn on the gene expression of TNF-α

After burn injury, the relative expression of TNF-α gene in the normal weight group, overweight and obese groups increased gradually on days 1 and 3, and peaked on day 7. Factorial analysis showed that burn and obesity factors had an interactive effect on the expression of TNF-α gene on days 1 and 3, when the level of TNF-α in the obese group was higher than the overweight and normal weight groups (*P* < 0.05). Severe burns had a tendency to increase the expression of TNF-α in the obese group. The mean level of TNF-α was highest in the obese weight group, with the overweight group expressing more than the normal weight group (*P* < 0.05) (Table 4, Figs. 3C, 4C).

**Table 4:** The effect of burns and obesity on the relative expression of TNF- and VEGF gene in wounds.

Factor	Test days	df	TNF-α		VEGF	
			*F*	*P*	*F*	*P*
Burn severity	1	1	6.283	0.019	3.189	0.087
	3	1	3.595	0.070	15.933	0.001
	7	1	12.310	0.002	10.607	0.003
	14	1	0.590	0.450	1.909	0.180
Degree of obesity	1	2	27.551	0.000	5.370	0.012
	3	2	13.324	0.000	19.374	0.000
	7	2	4.510	0.022	18.223	0.000
	14	2	5.999	0.008	4.938	0.016
Burn severity^*^ Degree of obesity	1	2	10.516	0.001	0.007	0.993
	3	2	3.796	0.037	18.873	0.000
	7	2	0.132	0.877	9.545	0.001
	14	2	0.015	0.985	3.391	0.050

**Notes.**

Factorial analysis showed that obesity and burn severity had an interactive effect on TNF-α and VEGF relative expression, so the interaction between factors Burn severity * Degree of obesity was included in the analysis. There was no need to analyze the effect of burn severity and obesity separately, as they were both in the model.

#### The effect of obesity and burn on the gene expression of VEGF

Factorial analysis showed that burn and obesity factors had an interactive effect on the expression of VEGF gene on days 3, 7 and 14. On day 3 after burns were inflicted, the level of VEGF was higher in the normal weight group compared to the obese group (*P* < 0.05). On day 7, the level of VEGF in the overweight group was higher than that in the normal and obese weight groups. Compared with the rats with second-degree burns, the level of VEGF in overweight and normal weight groups in rats with third-degree burns showed a decreased expression, and the obese group showed an increase in VEGF. On day 14, increased burn severity tended to decrease the expression of VEGF in the obese and normal weight groups, and increase VEGF expression in the overweight group. The mean level of VEGF was highest in the normal weight group, with the overweight group expressing more than the obese group (Table 4, Figs. 3D, 4D).

## Discussion

Adipose tissue is an active endocrine organ that secretes a series of biologically active molecules, including MCP-1, IL-1, IL-10, and TNF-α, also known as adipocytokines (Joffe, Collins & Goedecke, 2013). Studies have shown that MCP-1 is significantly increased in adipose tissue of obese rats. Over-expressed MCP-1 increases macrophage infiltration in adipose tissue. Macrophages secrete the potent pro-inflammatory cytokines such as TNF-α, IL-1, IL-10 and MCP-1, leading to further inflammation (Boutens & Stienstra, 2016; Cranford et al., 2016; Remmerie, Martens & Scott, 2020).

Burn wounds initiate a metabolic process by releasing catecholamines, growth hormones and cytokines. The body initiates an innate immune response to burns. Neutrophil is exuded in the wound tissue, and monocytes and macrophages produce a large number of pro-inflammatory cytokines, leading to an excessive inflammatory response. TNF-α, IL-1, IL-6, and IL-8 are important inflammatory cytokines responding to burn wounds. In the inflammatory response, TNF-α can promote the differentiation of bone marrow cells into monocytes and macrophages. TNF-α, IL-1, and IL-8 can also increase vascular permeability, procoagulant activity of vascular endothelial cell (VEC), and increase the adhesion to neutrophils (Roy & Sen, 2012). The inflammatory reaction is the inevitable process of wound healing. A variety of immune cells (macrophages, neutrophils, endothelial cells, etc.) and immune molecules are involved, mediating the immune defense function of cells, and jointly eliminating the source of infection, thereby promoting wound repair and healing (Larouche et al., 2018; Salibian et al., 2016; Stone et al., 2018). As a chemokine, MCP-1 chemically signals macrophages to arrive at the site of inflammation, mediating the inflammatory response and playing an important role in wound repair. Low et al. (2001) found that removing MCP-1 genes, significantly reduced angiogenesis and collagen synthesis. Chemokines promote wound healing mainly by promoting the formation of VEC to promote granulation tissue growth and scar formation. Chemokines interact with inflammatory factors such as TNF-α and IL-1 to induce VEGF and PDGF in wound keratinocytes and macrophages (Larouche et al., 2018; Ridiandries, Tan & Bursill, 2018; Thomas & Apovian, 2017). VEGF can specifically act on vascular endothelial cells, and has the function of maintaining vascular integrity, improving vascular permeability, and promoting angiogenesis (Cai et al., 2017; Komi, Khomtchouk & Santa Maria, 2020).

Serum ELISA protein assays showed that in the obese and over-weight group, the MCP-1, IL-1β and TNF-α protein expression were higher than normal in the unburn control group, suggesting that obese and overweight rats were in a slightly inflamed state, and the result is similar to the research of Wang et al. (2018). Ziraldo et al. (2013) found that patients with higher MCP-1 in the serum had longer hospital stays and time of required mechanical ventilation, and usually had a poorer prognosis. In our study, the effect of burn on the expression of MCP-1, IL-1β and TNF-α protein in serum was not obvious. It may be because the burn area of our experiment was not large. The wound area was 10 cm^2^, which was not enough to cause the protein expression changes of MCP-1, IL-1β and TNF-α in the blood.

The RT-PCR results of wound tissue showed that the relative expressions of MCP-1, IL-1β and TNF-α genes were highest in the obese group, which was in line with previous research that obesity poses a risk of inflammatory cytokines expression (Boutens & Stienstra, 2016; Remmerie, Martens & Scott, 2020; Cheng, 2016). Additionally, this research showed severe burns have a tendency to promote the expression of MCP-1, IL-1β and TNF-α genes in the obese group. It suggests that obesity combined with severe burns have a severe inflammatory response compared with normal weight and overweight groups. Obesity has a pro-inflammatory effect in rats with burn injuries. On days 3 and 7 after inflicting burns, the expression of VEGF in normal weight second-degree burn group was higher than that in overweight and obese groups. The expression of VEGF gene in normal and overweight groups decreased after third-degree burns were inflicted, suggesting that VEC regeneration was reduced after severe burns. On the 14th day, the obese and normal weight groups with severe burns had decreased VEGF expression, however VEGF increased in the overweight group, suggesting that the regeneration of endothelial cells in overweight rats recover better from severe burns.

## Conclusion

In this study, a factorial design was used to investigate the changes of several inflammatory factors and growth factor VEGF in blood serum and wound tissue of burned rats with different degrees of obesity. This study showed that the relative expression of MCP-1, IL-1β and TNF-α genes in the wounds of the obese group was higher, leading to a larger inflammatory reaction compared to the normal weight group. It suggested that obesity could promote the inflammatory response caused by burns. Obesity has a pro-inflammatory effect in rats inflicted with burns. This study does not examine the effect of obesity on the wound healing rate. Future research needs to explore the different healing rates of burn wounds with and without obese weight conditions.

##  Supplemental Information

10.7717/peerj.10499/supp-1 Supplemental Information 1 Dataset

## Additional Information and Declarations

### Data Availability

The following information was supplied regarding data availability: The raw measurements are available in the Supplementary File.
